# Epigenetic regulation as a therapeutic target in the malaria parasite *Plasmodium falciparum*

**DOI:** 10.1186/s12936-024-04855-9

**Published:** 2024-02-12

**Authors:** Thibaud Reyser, Lucie Paloque, Jean-Michel Augereau, Luisa Di Stefano, Françoise Benoit-Vical

**Affiliations:** 1 LCC-CNRS, Laboratoire de Chimie de Coordination, CNRS, Université de Toulouse, Toulouse, France; 2 MAAP, Inserm ERL 1289, Team “New Antiplasmodial Molecules and Pharmacological Approaches”, Toulouse, France; 3 Institut de Pharmacologie et de Biologie Structurale, IPBS, CNRS, UPS, Université de Toulouse, Toulouse, France; 4 MCD, Centre de Biologie Intégrative (CBI), CNRS, UPS, Université de Toulouse, Toulouse, France

**Keywords:** *Plasmodium*, Epigenetics, Histone modifications, Epidrugs, Antiplasmodial drugs

## Abstract

Over the past thirty years, epigenetic regulation of gene expression has gained increasing interest as it was shown to be implicated in illnesses ranging from cancers to parasitic diseases. In the malaria parasite, epigenetics was shown to be involved in several key steps of the complex life cycle of *Plasmodium*, among which asexual development and sexual commitment, but also in major biological processes like immune evasion, response to environmental changes or DNA repair. Because epigenetics plays such paramount roles in the *Plasmodium* parasite, enzymes involved in these regulating pathways represent a reservoir of potential therapeutic targets. This review focuses on epigenetic regulatory processes and their effectors in the malaria parasite, as well as the inhibitors of epigenetic pathways and their potential as new anti-malarial drugs. Such types of drugs could be formidable tools that may contribute to malaria eradication in a context of widespread resistance to conventional anti-malarials.

## Background

According to the latest World Health Organization (WHO) malaria report, an estimated 608,000 people died from malaria in 2022 [1]. Even though malaria cases are significantly lower than in 2000, and after years of increases until 2019, the number of deaths is falling again compared with 631,000 in 2021 [1]. Artemisinin-based combination therapy (ACT) has significantly helped to reduce malaria death toll since its introduction in 2001. However, emergence of artemisinin resistance in Southeast Asia threatens the use of artemisinin-based combinations. The worst fears of the scientific community [2, 3] are beginning to manifest as evidenced by the recent emergence of artemisinin resistance in Africa [4–6], where the parasite already kills 95% of its victims [7]. Therefore, widening the drug portfolio by identifying new anti-malarial drug targets is paramount. Among them, epigenetic mechanisms stand out because they are involved in the regulation of gene expression, which is closely linked to many key biological processes of the *Plasmodium* parasite [8]. Therefore, drugs targeting epigenetic pathways, or epidrugs, in the malaria parasite could be a winning strategy towards malaria eradication.

## Epigenetics: some generalities

Epigenetics which literally means ‘‘outside the genome’’, has become a very trendy field of research in the past twenty years. However, its origin dates back to the 1940s, when Conrad Waddington coined the term of epigenotype to describe the link “between genotype and phenotype (that) lies a whole complex of development processes” [9]. Epigenetics was more precisely defined as the study of the mechanisms regulating gene expression without causing any change in the DNA sequence while being transmissible to the following generations [10, 11]. The most studied epigenetic mechanisms, mainly histone post-translational modifications (HPTMs) and DNA modifications, are thus involved in controlling the accessibility of the coding genetic sequence to transcriptional effectors, and depend on three different sets of proteins: writer proteins that deposit modifications on histone or DNA, reader proteins that recognize and bind to the modifications and eraser proteins able to catalyze the removal of the modifications [10]. It is important to keep in mind that these levels of regulation of gene expression overlap with several other processes such as RNA modifications, RNA degradation or non-coding RNA [10] challenging the strict association between specific epigenetic marks and gene expression/silencing.

### *Histone post-translational modifications*

**Fig. 1:**
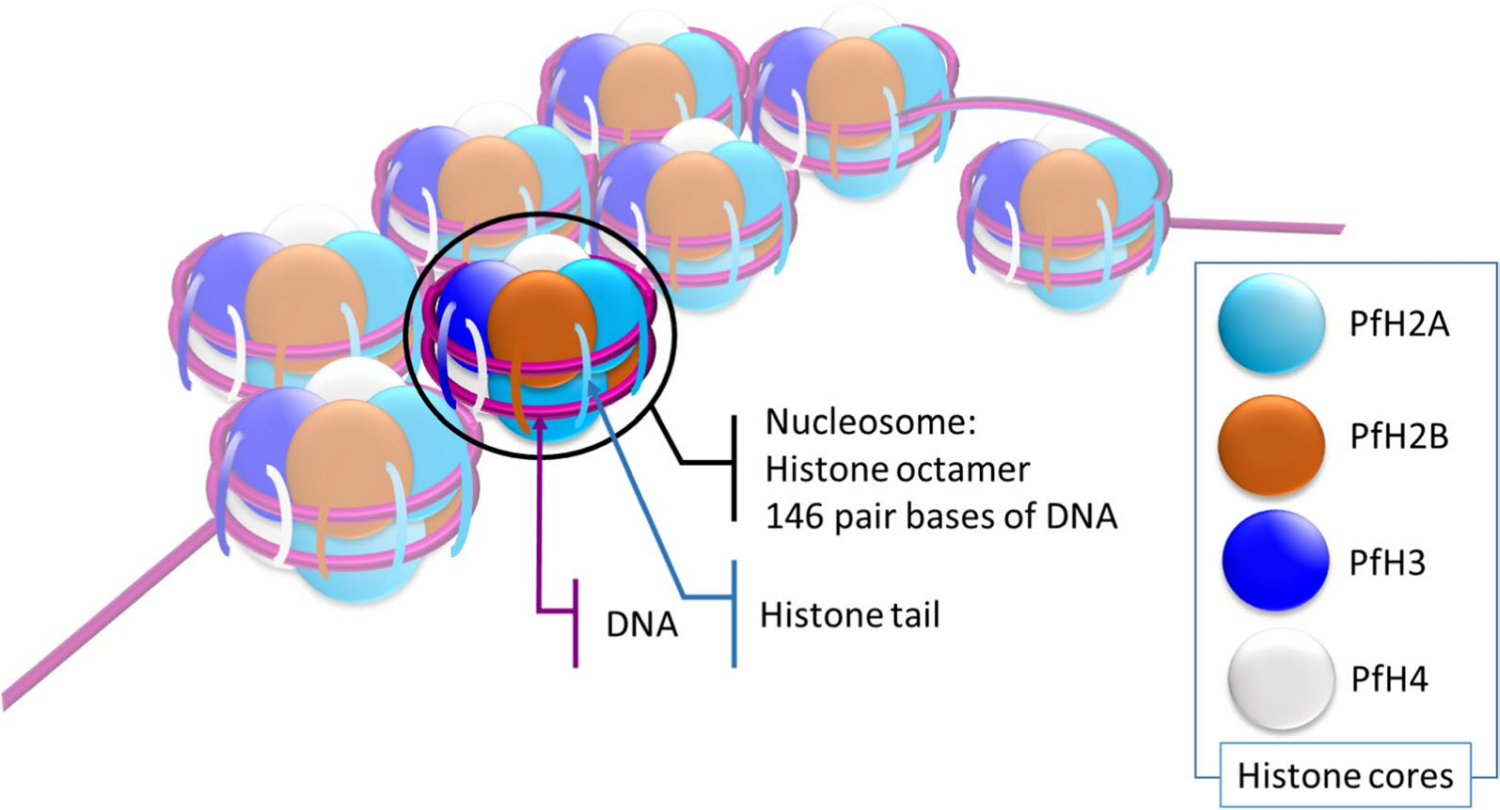
Schematic view of a *Plasmodium falciparum* nucleosome organization. DNA is wrapped up around nucleosome, a histone core octamer structure, made of two H2A-H2B dimers bound to an H3-H4 tetramer [12]. Histone tails and in particular lysine and arginine residues, on which several post-translational modifications can occur, are generally positively charged allowing strong binding to DNA which possesses negatively charged phosphate groups

DNA is wrapped up around histone proteins (Fig. 1), forming the basic structural unit of genome packaging, the nucleosome. Gene expression relies in part on the degree of compaction of chromatin, which depends on the state of the nucleosome. The histone protein is made up of two protein domains: a core structure whose main role is to mediate histone/histone interactions, and a N-terminal tail mediating histone/DNA electrophile interaction [12]. N-terminal tail of histones can also serve as a binding site for various proteins involved in chromatin remodeling, transcriptional regulation, and other cellular processes. These interactions can be modulated by the presence of specific post-translational modifications on the tail, allowing for dynamic and context-dependent regulation of gene expression. These post-translational modifications of histone tails are of several types and include acetylation, methylation, phosphorylation, ubiquitylation, citrullination, sumoylation, ADP-ribosylation, propionylation, butyrylation, formylation, proline isomerization and crotonylation of various amino acid [13, 14]. When nucleosomes are tightly packed, the chromatin is in a condensed state called heterochromatin, which was originally thought to prevent gene expression. When nucleosomes are less compacted, the chromatin is in a loose state called euchromatin, which allows regulatory proteins (including transcription factors) to easily access the DNA sequence and allow gene expression. However, it has been shown that gene expression can occur in heterochromatic domains, challenging the strict view of heterochromatin as a "silent" component of eukaryotic genomes [15].

**Fig. 2:**
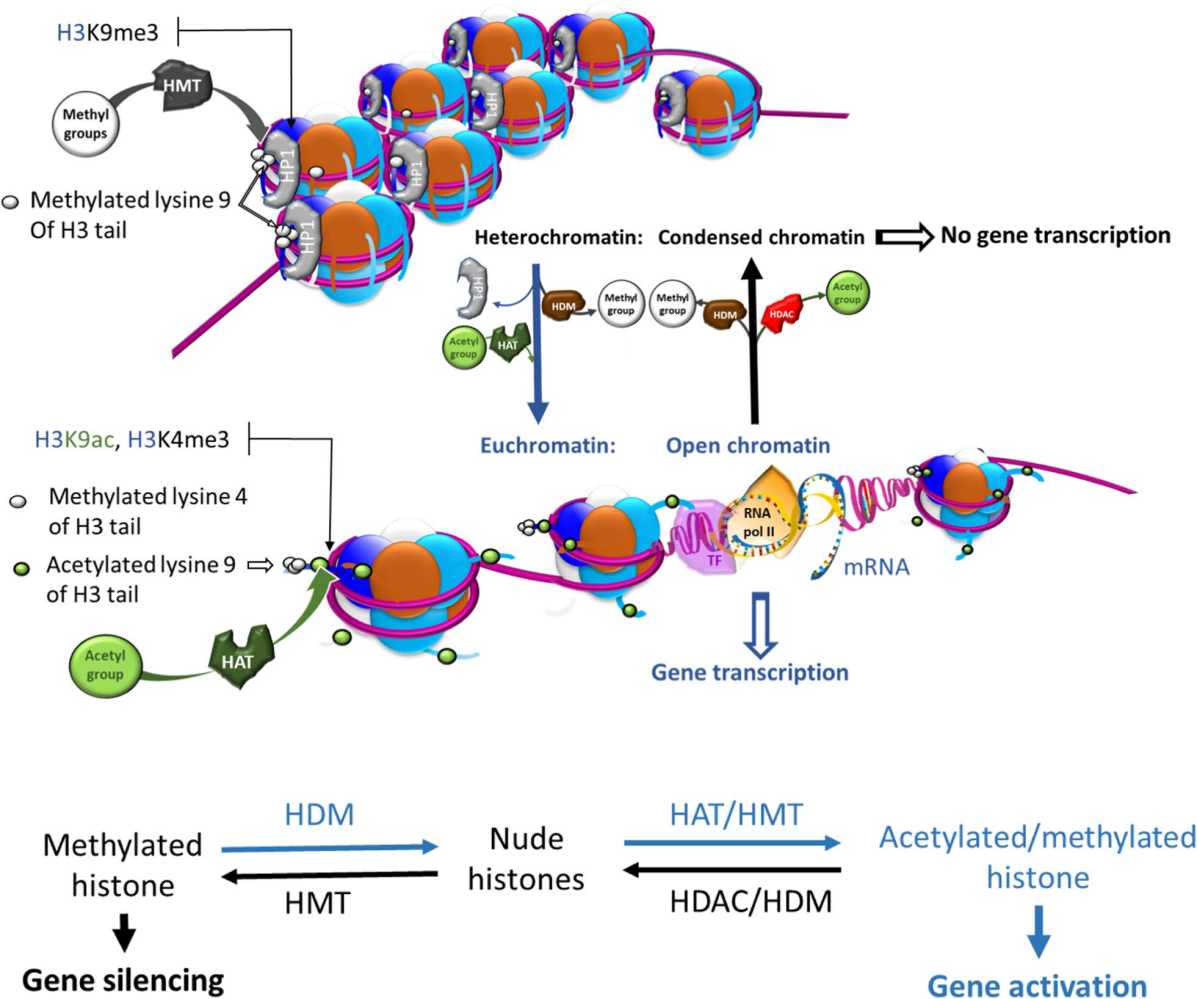
Relationship between acetylation and methylation levels for the switch from heterochromatin to euchromatin. Histone methylation as the tri-methylated of the 9th lysine residue of histone 3 tail, via histone methyltransferases (HMT), is globally involved in gene silencing. These modifications tend to recruit histone binding proteins (such as heterochromatin protein 1 (HP1)) that avoid chromatin relaxation, thus preventing transcription factors from accessing DNA. This methylation state is reversible and mediated by histone demethylases (HDM) [12, 22, 23]. However, histone methylation is not always associated with gene silencing. Trimethylation on the 4th lysine of histone 3 (H3K4me3) is involved in gene expression [17–19]. When a histone tail is acetylated by histone acetyltransferases (HAT), this tends to neutralize the lysine positive charge interacting with the negative phosphate groups of DNA and pushes away histone cores therefore “opening” the chromatin. That allows transcription factors (TF) to recognize and bind to promoters and RNA polymerase II (RNA pol II) to initiate transcription. This acetylation level is reversible and mediated by histone deacetylases (HDAC)

The two most largely studied histone modifications are histone tail acetylation (on lysine residues) and methylation on both lysine or arginine residues [13] mainly located on the N-terminal part of histones H3 and H4 [16] (Fig. 2). The addition of an acetyl moiety (CH3-CO-) on a positively charged lysine reduces the histone tail interaction with DNA leading to a more open chromatin conformation and, therefore, favouring gene activation. Conversely, the electronic charge of the amino-acid side chain is not altered by the methyl moiety (CH3), and histone methylation has been correlated to either gene repression or activation depending on the residue affected. Thus, trimethylation on the 9th lysine of histone 3 (H3K9me3) is abundant in heterochromatic domains and correlates with gene repression while trimethylation on the 4th lysine of histone 3 (H3K4me3) is abundant in euchromatin and correlates with active transcription [17–19]. It is thought that reader proteins are able to recognize the different states of methylation and acetylation [20]. Acetylation and methylation on lysine residues are mutually and dynamically exclusive for the same amino groups depending on the cell cycle development. Therefore, histone deacetylases/methyltransferases and histone demethylases/acetyltransferases are tightly linked in order to fine-tune gene expression [21].

### *DNA modifications*

**Fig. 3:**
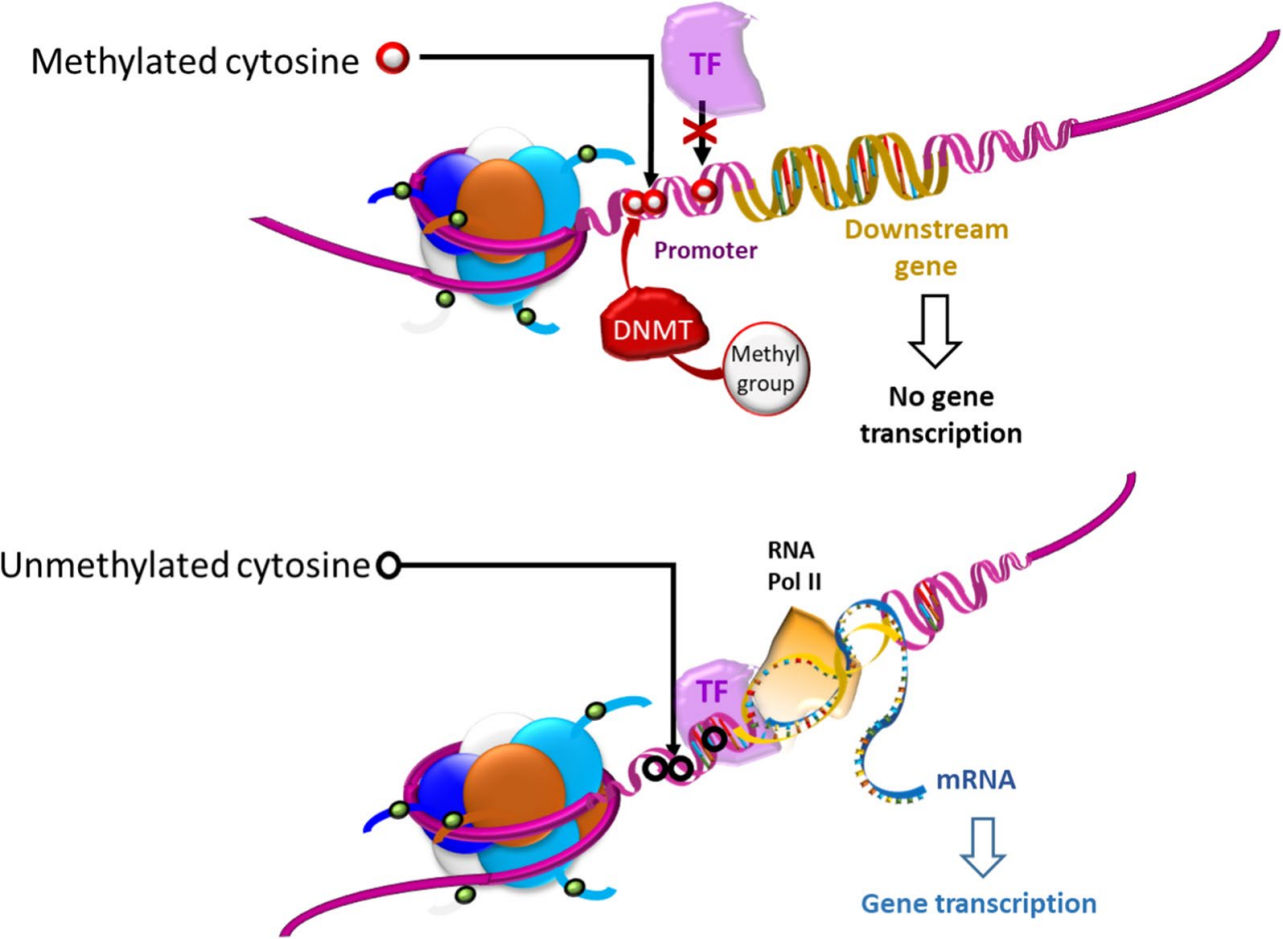
DNA methylation is mediated by DNA methyltransferases (DNMTs). Methylated cytosines have an impact on gene expression when they are located in promoter regions of genes. When present, this modification prevents transcription factors (TF) from binding to promoter regions and starting transcription and thus downstream gene expression. In the absence of DNA methylation (unmethylated cytosine), transcription factor can bind to the DNA strand and transcription of the gene concerned can proceed

DNA base modifications generally affect the accessibility of genomic regions for regulatory effectors of gene expression (Fig. 3). The most common modification consists in the addition of a methyl group to the carbon 5 of a cytosine (5mC) [24] and is generally associated with loss of gene expression [25]. DNA methylation is recognized and bound by specific methylated cytosine-binding proteins, which can in turn recruit co-repressor complexes. Through steric hindrance, these protein complexes could then prevent transcription factors from binding to promoter regions, thereby silencing downstream gene expression [26].

## Epigenetic regulation in the malaria parasite

In a general framework, regulation of gene expression occurs at multiple levels: basal (or constitutive) transcription is assured by general transcription factors while sequence-specific transcription factors bind to cis-regulatory regions of genes (enhancers, promoters) and allow individual genes to be turned on or off in specific cell types. Twenty-seven Apicomplexan-specific AP2 (ApiAP2) DNA-binding proteins have been identified in *Plasmodium falciparum* and they are the main factors regulating transcription. Although they are not as numerous as in other eukaryotic organisms with comparable genome sizes, such as yeast, their function is essential to the parasite's life cycle and its ability to adapt to changes in its environment [27–30]. PfAP2-P is involved in the regulation of gene expression during parasite development growth and pathogenesis [30] and PfAP2-G in the switch from asexual to gametocytes [31, 32]. Thus, in *Plasmodium,* epigenetic regulations could represent a main form of regulation of gene expression close to those in ancestral eukaryotic groups [33] at each step of the parasite life cycle, either at the intra-mosquito, hepatic or intraerythrocytic stage [34–37]. Epigenetics regulates key processes of *Plasmodium* biology (recently reviewed [8, 38]) such as: (i) immune evasion through the “one at a time” expression of clonally variant genes coding for surface antigen like PfEMP1 [39], (ii) the “just in time” regulation of gene expression required for the cell cycle progression during the intraerythrocytic stage [40], (iii) DNA repair mechanisms [41, 42], (iv) sexual commitment [43–46], or (v) adaptation to environmental changes [47]. While histone post-translational modifications in malaria parasites were described some 30 years ago [48, 49], DNA modifications in the parasite have been recently discovered, and although they are lowly abundant, their role in regulating the transcriptional state of the parasite genome is starting to be elucidated [50–52]. Two other epigenetic mechanisms, relying on RNA modifications [53, 54] and on non-coding RNAs [55] also exist in the malaria parasite. In this way it has been recently shown the importance of long non-coding RNAs in pathogenicity and sexual differentiation [56, 57].

### *Epigenetic marks in* Plasmodium

#### Histone post-translational modifications (HPTMs) in *Plasmodium*

At least 232 histone post-translational modifications have been identified in *P. falciparum* [58], including ubiquitylation and phosphorylation [59, 60], but the role of many of these marks remains unclear. *P. falciparum* has a very original epigenetic signature, with a significant number of activating histone marks yet only a handful of repressive marks. Indeed, activating histone marks are abundant and scattered throughout the genome, allowing the transcriptionally-permissive state of the genome along the intra-erythrocytic development cycle [60]. The number of HPTMs can vary, i.e., on average of 3 per histone tail but which can go up to 7 [61]. Among them, H3K9ac and H3K4me3 are the most abundant ones [62, 63] and their dynamic distribution is tightly linked with the “just in time” pattern of gene expression along the 48-h of the parasite intraerythrocytic developmental cycle, in other words only at a time it is required [40]. In this sense, a specific HPTM profile of gametocytes can also be seen, with a high abundance of acetylated histones H3 and H4 [64].

Indeed, highly transcribed genes of* P. falciparum* are associated with enriched H3K9ac marks in their promoter and 5’ coding sequences of active genes. H3K4me3 is stage-specific i.e. low at early stages, peaking at late stages, does not appear to be correlated with gene expression [65] [63] as it is not dynamically enriched at active promoters, but is upregulated at intergenic regions especially at trophozoite and schizont stages [63]. Repressive histone marks, such as H3K9me3, are specifically associated with clonally variant genes, such as *var*, *rifin* and *stevor,* that are localized on subtelomeric and some chromosome internal regions [66].

Other repressive marks include H3K36me2, H4K20me3 and H3K27me3, identified in gametocytes [67]. Moreover, it has been shown that phosphorylation of the histone H2A on serine 121 occurs in case of DNA damage before the DNA repair systems are activated and removed once the repair process has started [41].

#### DNA modifications in Plasmodium

For a long time, the existence of methylated DNA within the parasite was highly debated. After its identification in 2013, its level was estimated at 0.01–0.05% to 0.58% of genomic cytosines in *P. falciparum*. 5-methyl cytosines (5mC) were later identified in *Plasmodium berghei* [50–52]. Very recent data have shown that 5mC is in fact at a level of 0.1–0.2% during the intra-erythrocytic cycle [68] close to that of other apicomplexans, such as *Toxoplasma gondii* (0.27–0.41%) [69], but far behind that of mammals and birds (5%), fish (around 10%) or plants (as high as 30%) [70]. Hydroxy-methylated cytosines (5hmC) have also been identified and seem to be correlated with gene expression. They could represent 0.2 to 0.4% of genomic cytosines in malaria parasites, significantly more than the 0.03 to 0.06% in other organisms [50] but it is not confirmed in another study [68]. These DNA modifications could be also concomitant with histone marks [51], similar to what has already been seen in model organisms such as *Xenopus* [26].

### *Epigenetic effectors in* Plasmodium

Histone and DNA modifications are under the control of specific enzymes which are responsible for ‘writing’ and ‘erasing’ a wide range of modifications on histone tails among which acetylation and methylation are the most studied ones.

#### Histone acetyltransferases/histone deacetylases

Histone acetylation is catalyzed by histone acetyltransferases (HATs) (Table 1). Four different HATs have been identified in the *Plasmodium* genome: *Pf*HAT1, *Pf*ELP3, *Pf*GCN5 and *Pf*MYST [71], but only the activity of the last two was determined. *Pf*GCN5, is a nucleolar enzyme [72, 73] also active in the regulation of clonally variant gene expression [74]. It has recently been shown that *Pf*GCN5 can be found in different protein complexes especially in the later stages of the erythrocytic cycle of the parasite. Multiple variants of a *Pf*GCN5-containing complex could be capable of performing different biological functions [74]. *Pf*MYST is essential for gene expression, cell cycle progression and DNA repair [42] and its over expression entails a significant hyperacetylation at H4K5, K8, K12 and K16 which is associated with shortened intra-erythrocytic developmental cycle and reduced growth rate [75]. However, this HAT is not specific to histones since it also acetylates cytoplasmic proteins [42].

**Table 1:** Histone writers and erasers enzymes in *P. falciparum*

Name	PlasmoDB ID	Inferred/Known activity
Histone acetyltransferases (HATs)		
*Pf*GCN5 [72]	PF3D7_0823300	H3K9, H3K14 acetylation
*Pf*MYST [75]	PF3D7_1118600	H4K5, K8, K12, K16 acetylation
*Pf*HAT1 [71]	PF3D7_0416400	Unknown
*Pf*ELP3 [71]	PF3D7_1227800	Unknown
Histone deacetylases (HDACs)		
*Pf*HDAC1 (Class I) [81]	PF3D7_0925700	Parasite’s progression through intraerythrocytic developmental cycle
*Pf*HDA2 (Class II) [45]	PF3D7_1008000	H3K9 deacetylation involved in gametocyte commitment, virulence gene silencer
*Pf*HDA1 (Class II) [82]	PF3D7_1472200	Putative HDAC
*Pf*SIR2A (Class III) [85, 86]	PF3D7_1328800	Telomere maintenance and *var* gene silencing
*Pf*SIR2B (Class III) [88, 143]	PF3D7_1451400	Silencing of *var* genes
Histone methyltransferases (HKMTs) [90, 94, 144]		
*Pf*SET1	PF3D7_0629700	H3K4 methylation
*Pf*SET2	PF3D7_1322100	H3K36 methylation
*Pf*SET3	PF3D7_0827800	H3K9 methylation
*Pf*SET4	PF3D7_0910000	H3K36 methylation
*Pf*SET5	PF3D7_1214200	Unknown
*Pf*SET6	PF3D7_1355300	H3K36 methylation
*Pf*SET7	PF3D7_1115200	H3K36, H3K9, H3K4 methylation
*Pf*SET8	PF3D7_0403900	H4K20 methylation
*Pf*SET9	PF3D7_0508100	H3K36 methylation
*Pf*SET10	PF3D7_1221000	H3K4 methylation
*Pf*PRMT1	PF3D7_1426200	H4R3 methylation
Histone lysine demethylases [91, 96]		
LSD1 family		
*Pf*LSD1	PF3D7_1211600	H3K4me1 and me2 demethylation
*Pf*LSD2	PF3D7_0801900	H3K4me1 and me2 demethylation
Jumonji-related family		
*Pf*JmjC1	PF3D7_0809900	Histone Lysine Demethylase
*Pf*JmjC2	PF3D7_0602800	Histone Lysine Demethylase
*Pf*JmjC3	PF3D7_1122200	Histone Lysine Demethylase
DNA methyltransferase (DNMT) [51, 53, 101]		
*Pf*DNMT2	PF3D7_0727300	DNA and tRNA methylation

Histone deacetylation is mediated by histone deacetylases (HDACs) (Table 1) [49, 76, 77]. Five HDACs have been identified in *P. falciparum* and subdivided in 3 categories based on their phylogenetic relationship to their yeast orthologues [71]. Class I and Class II enzymes have a zinc-dependent HDAC activity and act on intra-chromosomal domains whereas class III HDACs are NAD + dependent and are involved in silencing genes in sub-telomeric regions [39, 78–80]. PfHDAC1 (class I) could be involved in the reversible changes of euchromatin mediating the intraerythrocytic developmental cycle of the parasite [81]. The class II includes PfHDA1 and PfHDA2 which are key players of gametocyte commitment and play a role in irreversible changes of chromatin structure involved in this key step of the life cycle [45, 82–84]. In class III, PfSir2A has the ability to deacetylate both histone 3 and histone 4. This SIR2 deacetylase activity is necessary for virulence gene silencing [85]. Conversely acetylation of histones, in particular H4, can occur when PfSIR2 is removed from the promoter region of the subtelomeric var gene [86]. PfSIR2A can be considered as a major var-associated deacetylase. Another histone deacetylase Sir2 has been identified, PfSIR2B which like PfSIR2A regulates silencing of *var* genes in *P. falciparum* but for a different subset [84, 87, 88]. Similarly to their orthologs in other eukaryotes, these two sirtuin enzymes might also play a role in the adaptation of the parasite to its environment [89].

#### Histone methyltransferases / histone demethylases

Methylation on histones can take place either on amino-groups of lysine or on guanido nitrogen atoms of arginine and is mediated by histone lysine methyltransferases (HKMT) or protein arginine methyltransferases (PRMT) (Table 1). Three putative PRMTs (*Pf*PRMT1, *Pf*PRMT5, *Pf*CARM1), most likely involved in protein maturation than regulation of gene expression, have been identified in *Plasmodium* but only *Pf*PRMT1 has been characterized [90] and recently *Pf*PRMT5 which plays a key role in merozoite invasion [56]. Ten *Pf*HKMTs (also known as SET1 to SET10) have been identified by computational analysis [91, 92]. It should be noted that these proteins do not only methylate histones but a large range of proteins, which can be found either in the nucleus or the cytoplasm. *Pf*SET10 may play a role in the regulation of *var* genes expression through its ability to methylate the lysine K4 of histone H3 [92], but this remains a matter of debate [93].

Histone demethylation is mediated by histone lysine demethylases (HKDM) (Table 1). Five HKDMs have been identified and sub-categorized into two categories: LSD1 and Jumonji (JmjC) demethylases [91, 94]. While LSD1 demethylases can be involved in the removal of mono- and dimethylated groups from lysines [95], JmjC demethylases (*Pf*JmjC1, *Pf*JmjC2 and *Pf*JmjC3) are the only family capable of the demethylation of trimethylated lysine residues like H3K4me3, H3K9me3 and H4K20me3 in the parasite [96]. As previously mentioned, acetylation and methylation patterns of histones are linked. For example, sexual commitment regulation relies on a switch of H3K9me3 to H3K9ac depending on *Pf*SET3 and *Pf*GCN5 [97, 98]. This leads to the dissociation of H3K9–HP1 (heterochromatin protein 1) complex and the subsequent triggering of parasite commitment to gametocytogenesis through de-repression of *pfap2-g* [99, 100]. The return to a silencing state of this transcription factor depends on *Pf*HDA2 responsible for deacetylation of H3K9ac tail prior to its methylation [45].

#### DNA methyltransferases

Only one gene with a predicted DNA methyltransferase activity has been identified in *P. falciparum* genome (*Pf*DNMT2) coding for an enzyme related to the DNA methyl transferase 2 family but with a low methylation activity on DNA cytosines in vitro [51, 53] (Table 1). Expressed all along the intraerythrocytic cycle with a peak at the trophozoite stage, *Pf*DNMT2 is able to methylate tRNA cytosine including C38 of tRNA^Asp^ [68, 101]. The tRNA methylation participates in maintaining stable protein synthesis by protecting tRNAs from endonucleolytic degradation during stress situations experienced by the parasite [53].

## *Plasmodium* epigenetic effectors as a source of therapeutic targets

### Case of parasite resistance to anti-malarials

**Fig. 4:**
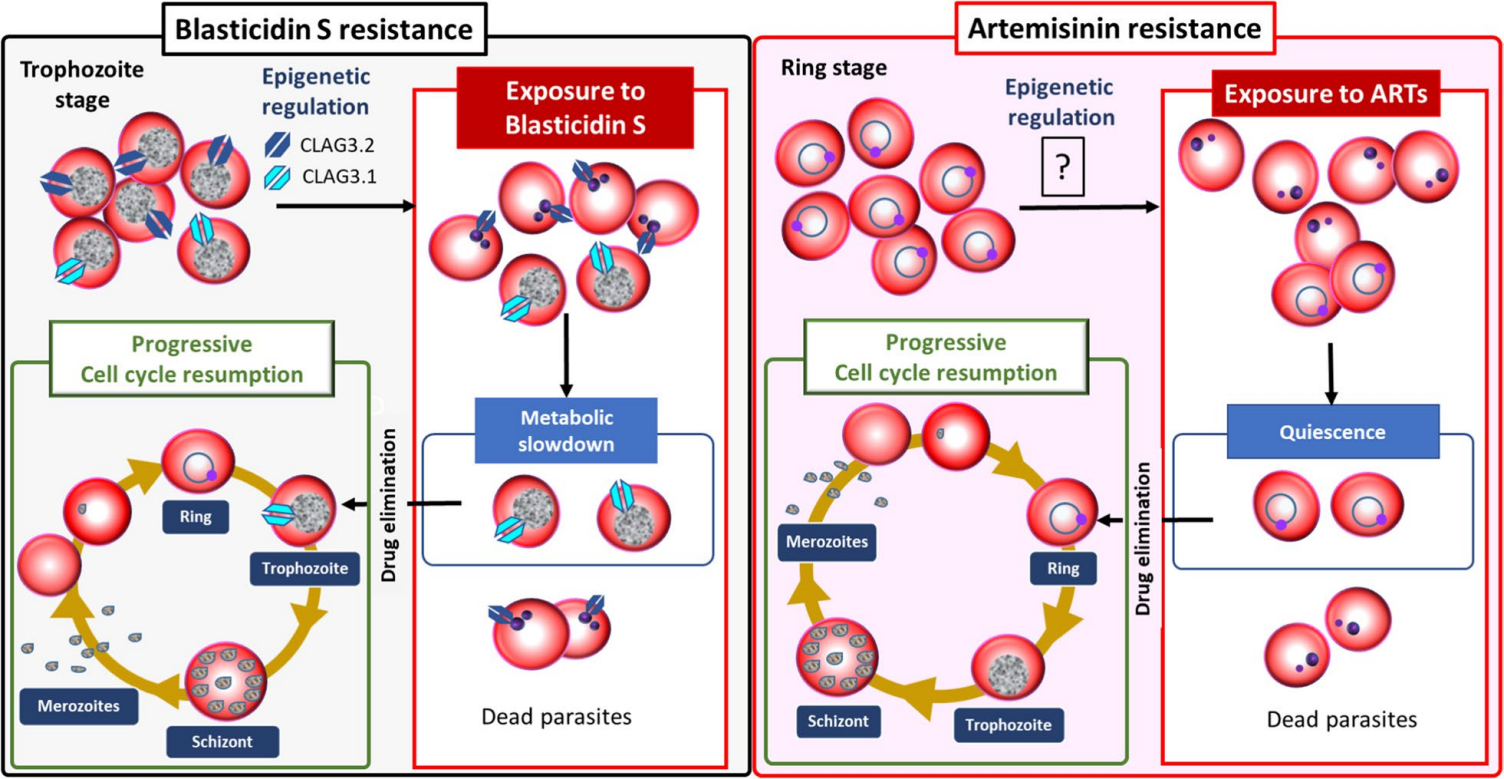
Role of epigenetics in the acquisition of drug resistance, a parallel between resistance to blasticidin S and artemisinin resistance. Upon the exposure of a trophozoite population to Blasticidin S, a majority of them dies because most of them express the CLAG3.2 protein at their cell surface. Only a subset of parasites expresses the CLAG3.1 protein, which allows them to withstand drug exposure at the cost of a slower metabolism. This regulation of CLAG3 genes is epigenetically-mediated, allowing a stochastic number of parasites to survive drug pressure [102, 103]. Following exposure to artemisinins, a subset of ring-stage parasites can enter a quiescent state by slowing down their metabolism. After drug removal, the parasites can resume their life cycle [110]

The first demonstrations that epigenetics could be involved in anti-malarial drug resistance processes in *Plasmodium* was obtained in relation to the antibiotic blasticidin S [102, 103] and the bis-thiazolium salts T3 and T16 [104]. Blasticidin S, an antibiotic, with an IC_50_ of 530 nM against the *Plasmodium* 3D7 strain, and T3 and T16 with IC_50_ values of 26 and 10 nM, respectively, all enter into the parasite through the solute transporter plasmodial surface anion channel (PSAC) [104]. This transporter is formed by a CLAG3 protein, either CLAG3.1 or CLAG3.2 with different solute uptake efficiency [47, 105]. Within a clonal population, *clag3.1* and *clag3.2*, localized head-to-tail in the same locus, are stochastically and mutually exclusively expressed in each parasite. When one is expressed, depending of H3K9ac, the other one is repressed (marked with H3K9me3) [106, 107]. The subset of parasites stochastically expressing no *clag3* gene or only CLAG3.1 (which has a low solute uptake efficiency) is able to withstand exposure to blasticidin S and T3 illustrating how epigenetics can mediate drug resistance in *Plasmodium* within isogenic parasite population where only few individuals can survive to the drug exposure [102, 104]. This is reminiscent of parasite resistance to artemisinins, since only a subpopulation within a clonal population, mutated for the *pfk13* gene involved in the endocytosis of haemoglobin from the host cell by the parasite [108, 109], can resist exposure to these anti-malarial drugs by entering quiescence [110, 111] (Fig. 4). It has also recently been shown that PfGCN5, a histone acetyl transferase, is involved in the resistance of *P. falciparum* to artemisinins by increasing the unfold protein response pathway (UPR) and controls 300–400 genes involved in stress responses [112, 113]. On this basis, it could be hypothesized that resistance to artemisinins in *P. falciparum* may also rely on epigenetic regulation.

Given the alarming increase in resistance to known available treatments finding new anti-malaria compounds is urgent. It has already been shown for many years that apicidin is able to inhibit histone modifying enzymes such as HDACs in all the stages of the *P. falciparum* intraerythrocytic cycle leading to parasite death [35, 49]. Moreover, chaetocin, a histone methyl transferase inhibitor, is able to reverse blasticidin S resistance [114]. Targeting histone modification thus appears to be an effective way to eliminate the malaria parasite, including resistant forms associated with diverse resistance mechanisms. Therefore, epigenetic drugs are very promising candidates because they target both the mechanisms of adaptability of the parasite to variations in its environment and its cell cycle regulation system. Interestingly, this approach can be extended at any stage of the parasite development cycle since epigenetics plays a crucial role throughout the parasite life cycle.

### Targeting histone modification

#### Targeting histone acetyltransferases/histone deacetylases

Inhibition of *P. falciparum* HATs has been largely described with anacardic acid, curcumin and embelin, resulting in hypoacetylation of lysine residues (Table 2). However, these inhibitors are highly unspecific since they can have many other effects such as on lipoxygenase activities, reactive oxygen species production, disruption of chaperone expression [115, 116]. At the opposite CB3717 was identified as showing strong selective inhibition of *Pf*GCN5 (which differs strongly from its human orthologue [74]) leading to a decrease of H3 acetylation level at K9 position. This compound with an IC_50_ at 200 nM range in parasite growth assay is tenfold less active against human cancer cells and shows no effect against mammalian fibroblast cells up to 20 µM [117]. *Pf*MYST is potentially another interesting target as it also differs significantly from its human orthologue and NU9056, a thiazole derivative, inhibiting *Pf*MYST catalytic activity is lethal for the parasite at a micromolar range [118] (Table 2). Although few *Plasmodium* HAT inhibitors have been described to date, new compounds designed to target HATs, involved in different pathologies, remain to be evaluated on the parasite and may provide interesting chemical starting points [119].

**Table 2:** Histone acetyltransferases (HAT) inhibitors in *P. falciparum*

Inhibitors	Structure	*P. falciparum* IC_50_ in µM	Specificity	CC_50_ on mammalian cells in µM (Cell type)
Anacardic Acid		30 [115]	Targets lipoxygenase activity [116]	> 100 (HeLa) [145]
Curcumin		25 [97]	No specificity, targets all HATs and involved in production of ROS [97]	4 (HL60) [146]
Embelin		10 [73]	Specific towards GCN5	> 40 (mammalian) [73]
CB3717		0.225; 1 [117]	Specific towards PfGCN5	> 20 (NIH3T3) [117]
NU9056		0.9 [118]	Specific towards PfMyst	-

Among antiplasmodial epidrugs, HDAC inhibitors are the most numerous with a wide variety of chemical structures (cyclic tetrapeptides, 2-aminosuberic acid derivatives or L-cysteine derivatives) (Table 3). Most of them were initially designed to target human cancer cells, and were later found to have high activities against *P. falciparum* with IC_50_ values ranging from low nanomolar to sub-micromolar but low selectivity for most of them [120, 121]. SAHA and CTP-NPDG 19, a cyclic tetrapeptide like apicidin display better activities against *P. falciparum* than towards cancer cells but the selectivity index remains weak [78, 122]. Use of HDAC inhibitors, such as trichostatin A and apicidin results in a significant increase in H4K8ac and H4Ac4 levels across the *Plasmodium* genome, both in asexual and sexual stages of *Plasmodium* [35, 123]. These changes in histone marks lead in turn irreversibly to a collapse of the tightly regulated transcriptional cascade in the early hours of drug exposure and ultimately to parasite death upon longer exposures [35, 122]. FNDR-20123, a hydroxamate derivative like SAHA and trichostatin A, appears to be a very promising HDAC inhibitor in a nanomolar range, with a good PK/PD and excellent safety profile [124]. Derivatives of the clinical anticancer drug candidate quisinostat, such as JX21108, a *Pf*HDAC1 inhibitor, present a good antimalarial activity and promising selectivity in vitro as in vivo in the *P. yoelli* mouse malaria model [125, 126](Table 3).

**Table 3:** Histone Deacetylase (HDAC) inhibitors in *P. falciparum*

Targeting Class I and II HDACs					
Inhibitors	Structure	*P. falciparum* IC_50_ in µM	Specificity	Development stage	CC_50_ on mammalian cells in µM (Cell type)
Cyclic tetrapeptides					
Apicidin		0.03; 0.04 [76, 147]	Targets class I and II HDACs [76]	–	10 (Jurkat) [148]
CTP-NPDG 19		0.3 [122]	Targets class I HDACs	–	> 25 (HepG2) [122]
Romidepsin		0.09 [149]	Targets class I and II HDACs	FDA-approved in cancer therapy	0.001 (NFF) [149]
HC-toxin		< 0.01 [49]	Targets class I and II HDACs	–	0.9 (TFK-1) [150]
FR235222		0.01 [151]	Targets class I HDACs	–	0.13 (HFF) [152]
Hydroxamates					
Trichostatin A		0.03; 0.08 [122, 147]	Targets class I and II HDACs	–	0.2 (HeLa) [153]
SAHA (Vorinostat)		0.2; 0.5 [76, 147]	Targets class I and II HDACs	FDA-approved in cancer therapy	5.5 (NFF) [149]
JAHA		0.5 [147]	Targets class I and II HDACs[154]	–	2.4 (MCF7) [154]
SBHA		1 [120]	Targets class I and II HDACs	–	12 (HEK) [155]
WR301801		0.001 [156]	Targets class I and II HDACs	Tested in vivo	0.6 (RAW) [156]
Belinostat		0.06 [149]	Targets class I and II HDACs	FDA-approved in cancer therapy	2.37 (NFF) [149]
Panobinostat		0.01 [149]	Targets class I and II HDACs	FDA-approved in cancer therapy	0.07 (NFF) [149]
FNDR-20123		0.04 [124]	Targets class I HDACs	–	> 100 (HepG2 & THP-1) [124]
JX21108		0.004 [125]	Targets class I HDACs	–	> 4 (HepG2) [125]
Compound 29		0.45 [139]	Targets class I HDACs	–	17 (Hela) [139]
Peptoid-based hydroxamic acids 2h		0.005 [138]	HDACs	–	4.6 (HepG2) [138]
2-aminosuberic acid derivatives					
2-aminosuberic acid derivatives		0.01; 0.3 [120]	Targets class I HDACs	–	0.2–5.8 (NFF) [120]
L-cysteine derivatives					
L-cysteine derivatives		0.05; 0.34 [120]	Targets class I HDACs	–	0.35–2.2 (NFF) [120]
Targeting Sir2 HDAC (Class III)					
Surfactin		9.3 [79]	Unknown off-targets since it induces apoptosis	–	9.6 (MCF-7) [157]
Sirtinol		10 [120]	No off-targets known Inhibits both PfSir2A and B	–	> 25 (NFF) [120]

#### Targeting histone methyltransferases/histone demethylases

The inhibition of HKMTs and HKDMs has been significantly less studied than the inhibition of HATs or HDACs in *P. falciparum*, probably due to their late identification. Nevertheless, some HKMT inhibitors have shown promising results with largely better selectivity index than the above-mentioned molecules and have gone so far as to be tested in vivo [127, 128] (Table 4). BIX-01294 and TM2-115 were shown to affect particularly H3K4me3 [127]. Although TM2-115 and BIX-01294 had a long-lasting effect on both *P. berghei* and *P. falciparum* parasites in mice models, they did not completely cure mice. Because of their oral bioavailability and their rapid ability to kill parasites, they nevertheless represent a good starting point for further development of the diaminoquinazoline compound series [128]. TM2-115 was also shown to activate dormancy exit of hypnozoites in *Plasmodium vivax* [129]. Collectively, these results suggest that HKMT inhibitors are very promising since they seem to target most of the life-cycle stages of the parasite. A medicinal chemistry approach could help to improve their efficacy and their pharmacokinetic profiles. There are currently few reports of drug discovery efforts specifically targeting arginine methylation in *P. falciparum* [90]. The search for new chemical starting points by an orthologue approach is not always successful as a recent study has shown on the evaluation of human histone demethylase inhibitors on their *P. falciparum* counterparts [130](Table 5).

**Table 4:** Histone methyltransferase (HKMT) inhibitors in *P. falciparum* targeting lysine methylation

Inhibitors	Structure	*P. falciparum* IC_50_ in µM	Specificity	Development stage	CC_50_ on mammalian cells in µM (Cell type)
UNC0638		0.028 [130]	Targets G9a methyltransferases in mammalian cells	–	23 (HL-60) [158]
BIX-01294		0.056; 0.075 [127, 147]	No target properly identified but inhibits H3K4me3	Tested on a humanized mouse model	6.1 (HFF) [127]
TM2-115		0.130; 0.137 [127, 147]	Identical to BIX-01294	Tested on a humanized mouse model	5.7 (HFF) [127]
Chaetocin		0.64; 0.95 [147, 159]; 14 [90]	No target properly identified but inhibits H3K9me2/me3	–	0.13 (HL-60) [158]

**Table 5:** Histone Demethylases (HKDM) inhibitors in *P. falciparum* targeting lysine demethylation

Inhibitors	Structure	*P. falciparum* IC_50_ in µM	Specificity	Development stage	CC_50_ on mammalian cells in µM (Cell type)
Tranylcypromine		> 10 [160]	Inhibits human LSD1 [161]	FDA-approved monoamine oxidase inhibitor	> 200 (HEK293) [162]
GSK-J1		> 10 [130]	Inhibits human Jumonji demethylases	–	9 (Human primary macrophages) [163]
IOX 1		1; 10 [160]	Inhibits human Jumonji demethylases [164]	–	86.5 (HeLa) [164]
JIB-04		0.6; 1.6; 0.47 [96, 147, 160]	Inhibits human Jumonji demethylases [96, 165]	–	> 10 (Human mesenchymal stem) [166]

### Targeting DNA modifications

For a time, targeting DNA methylation was generally overlooked in *P. falciparum*, mostly because of its low abundance [50]. Recently however, several quinazoline derivatives identified as human DNMT3 inhibitors were found to be active in vitro against *P. falciparum* and in vivo against *P. berghei* infected mice [131]. In a same way, series of quinoline-quinazoline bisubstrate analogues (Table 6), with an inhibitory activity towards human DNMT3a and DNMT1, has shown promising activities in the nanomolar range on *P. falciparum* strains [132].

**Table 6:** DNA Methyltransferares (DNMT) inhibitors in *P. falciparum*

Inhibitors	Structure	*P. falciparum* IC_50_ in µM	Specificity	Development stage	CC_50_ on mammalian cells in µM (Cell type)
5-azacytidine		1.5.10^3^ [51]	Human DNMT [167]	FDA-approved in cancer therapy [167]	2.3 (KG-1a acute myeloid leukemia)[168]
Decitabine		8.10^2^ [51]	Human DNMT[167]	FDA-approved in cancer therapy [167]	0.4 (KG-1a acute myeloid leukemia) [168]
SGI-1027		0.063; 0.051 [147, 160]	Human DNMT 1, 3A, 3B [169]	-	> 50 (H4IIE rat hepatoma) [169]
Quinoline-quinazoline derivative		0.06 [132]	*Pf*DNMT	Tested in standard chemosensitivity assays on ART-resistant strains	2.5 (HepG2) [132]
Quinazoline derivative		0.019 [131]	*Pf*DNMT3	Tested in standard chemosensitivity assays on multidrug resistant strains	> 1 (Splenic) [131]

### Targeting transcription factors

The twenty-seven main factors regulating transcription in *P. falciparum* can also be considered as very interesting targets. Their binding domains are different from human homologs which can be a guarantee of specificity for an inhibitor of these transcription factors. In silico prediction combined with biochemical and genetic studies have led to the identification of compounds with sub-micromolar antiparasitic properties, demonstrating the relevance of this approach [29].

### Epidrugs: a promising future?

Despite the fact that seven epidrugs such as DNMT (5-azacytidine) and HDAC inhibitors (SAHA) [133, 134] have been approved by government agencies (*e.g.* the FDA) in cancer therapies, lack of selectivity towards *Plasmodium* has been the major Achille’s heel in the development of antiplasmodial epidrugs. While a very good activity has been observed in vitro, in the nanomolar range on the parasite, the in vivo results in the *P. berghei* infected mouse model are often more than modest and administration of these compounds cannot cure the mice completely [135]. Nevertheless, as more and more efforts are being made to understand the mechanisms of epigenetic regulation of *P. falciparum*, the repositioning of anti-cancer drugs in the context of the search for new anti-malarial drugs remains very topical [125, 136].

In silico approaches, through molecular docking, also led to the discovery of a new portfolio of parasite specific HAT inhibitors [77, 117] and quantitative structure–activity relationship (QSAR) models have been developed as useful tools for in silico screening of *Plasmodium* HDAC inhibitors [137]. However, recent progress has been made in the field of malaria epidrugs in order to overcome potential toxicity issues in the mammalian host. SAR studies in vitro have led to the discovery of parasite specific compounds targeting HDACs and DNMTs with inhibitory values in sub-micromolar and nanomolar ranges and good selectivity indexes (> 100) [124, 132, 138, 139]. A recent review of inhibitors targeting *Plasmodium* HDACs and DNMTs lists the best compounds based on scaffold from a screening of epidrug libraries or from molecular docking studies. It highlights, in particular, the interest for in silico studies to optimize selectivity, pharmacokinetic properties and cost of goods [140].

Combining two different inhibitors in the same molecule is a very interesting approach, especially if they have a strong synergy. The hybrid compound procainainamide-SAHA, which combines a DNMT inhibitor (Procainamide) with an HDAC1 inhibitor (SAHA), has been shown to be highly active against *P. falciparum* multidrug-resistant field strains and lacks cytotoxicity against human cancer cells [141].

Therefore, epidrugs present several characteristics that are very desirable for an anti-malarial drug. Indeed, they can be fast-acting and could have high parasite-killing rates, probably due to the need for continual gene activation along the life-cycle stages. Pharmaco-modulation work has allowed notable increase of selectivity against *Plasmodium*. Moreover, due to the high conservation of histone modification enzymes and their assumed conserved role in transcriptional regulation across *Plasmodium* species, epidrugs are likely to be efficient on all human malaria pathogens, among which *P. falciparum* and *P. vivax* [72, 91, 142] and one could envisage a unique compound to treat all types of malaria. Epidrugs are very promising candidates because their interest lies in their ability to target both one of the major mechanisms of adaptability of the parasite to variations in its environment and its cell cycle regulation system. Whether during the intraerythrocytic cycle (pathogenicity), the induction of gametocytogenesis (transmission) or even in the DNA repair mechanisms following stress due to a change in the environment, epigenetics offers new opportunities for therapeutic targets covering all the different states of the parasite.

## Data Availability

Not applicable.
